# The Role of Neuropeptide Y in the Nucleus Accumbens

**DOI:** 10.3390/ijms22147287

**Published:** 2021-07-07

**Authors:** Masaki Tanaka, Shunji Yamada, Yoshihisa Watanabe

**Affiliations:** 1 Department of Anatomy, Kyoto Prefectural University of Medicine, Kawaramachi-Hirokoji, Kamikyo-ku, Kyoto 602-8566, Japan; syamada@koto.kpu-m.ac.jp; 2 Department of Basic Geriatrics, Kyoto Prefectural University of Medicine, Kawaramachi-Hirokoji, Kamikyo-ku, Kyoto 602-8566, Japan; y-watana@koto.kpu-m.ac.jp

**Keywords:** neuropeptide Y, nucleus accumbens, NPY receptors, fat intake, emotional behavior

## Abstract

Neuropeptide Y (NPY), an abundant peptide in the central nervous system, is expressed in neurons of various regions throughout the brain. The physiological and behavioral effects of NPY are mainly mediated through Y1, Y2, and Y5 receptor subtypes, which are expressed in regions regulating food intake, fear and anxiety, learning and memory, depression, and posttraumatic stress. In particular, the nucleus accumbens (NAc) has one of the highest NPY concentrations in the brain. In this review, we summarize the role of NPY in the NAc. NPY is expressed principally in medium-sized aspiny neurons, and numerous NPY immunoreactive fibers are observed in the NAc. Alterations in NPY expression under certain conditions through intra-NAc injections of NPY or receptor agonists/antagonists revealed NPY to be involved in the characteristic functions of the NAc, such as alcohol intake and drug addiction. In addition, control of mesolimbic dopaminergic release via NPY receptors may take part in these functions. NPY in the NAc also participates in fat intake and emotional behavior. Accumbal NPY neurons and fibers may exert physiological and pathophysiological actions partly through neuroendocrine mechanisms and the autonomic nervous system.

## 1. Introduction

Neuropeptide Y (NPY) is a highly conserved 36-amino-acid peptide that belongs to the same structural family as peptide YY and pancreatic polypeptide (PP) [1,2]. An abundant neuropeptide, NPY is widely distributed throughout the central nervous system of mammals [3]. In human and rodent brains, NPY mRNA and NPY-immunoreactive cell bodies and fibers are strongly expressed in regions such as the septum, nucleus accumbens (NAc), striatum, arcuate and paraventricular hypothalamic nuclei (PVN), cortex, amygdala, hippocampus, periaqueductal gray, and locus coeruleus [3,4,5,6,7,8,9,10]. NPY is also present in the peripheral nervous system, particularly the sympathetic nervous system, along with noradrenalin and adenosine triphosphate [11,12,13]. NPY in the peripheral nervous system is involved in cardiovascular control via sympathetic nerves during the acute phase of stress response [13]. Moreover, NPY has been found to exist outside the nervous system in a variety of peripheral tissues, such as retinal pigment epithelium, smooth muscle, intestine, bone marrow, and immune cells [14,15,16,17]. In these tissues, NPY acts as a modulatory factor on immune cells [18,19].

NPY is synthesized within the endoplasmic reticulum as a large precursor protein. After synthesis, it is moved to the Golgi apparatus and subsequently the trans-Golgi network, whereby the majority of peptide is stored in large dense-core vesicles [13,20]. Upon secretion of NPY into the extracellular space, the precursor protein undergoes several post-translational modifications along with several enzymatic cleavages [20]. The physiological and behavioral effects of NPY are mediated through several NPY receptor subtypes (Y1–Y5) [21,22,23,24]. Although the Y6 receptor has been reported in rabbits and mice [25], it is a non-functional pseudogene in humans [24]. The receptor originally identified as the Y3 receptor has since been characterized as CXC chemokine receptor type 4, a member of the chemokine receptor family [26]. The Y1 receptor was first cloned as an orphan receptor in mice [27], followed by cloning of the human Y2 receptor [28]. NPY shows strong affinity for Y1, Y2, and Y5 receptors [24], whereas the Y4 receptor prefers PP as a ligand to NPY [24]. The Y5 receptor was first cloned from rat hypothalamus, through which food intake was stimulated [29]. NPY receptors are Gi/o protein-coupled receptors and can therefore lead to hyperpolarization of the cell [30]. In general, NPY receptors mediate their actions postsynaptically, with the exception of Y2 receptors, which exhibit mostly presynaptic localization [31,32,33]. The Y1 receptor was also reported to exist in axon terminals [34]. Activation of NPY receptors primarily decreases cyclic adenosine monophosphate (cAMP) production by inhibiting adenylate cyclase in the cell [35]. In addition, NPY can lead to depressed Ca^2+^ channels and enhanced G protein-coupled inwardly rectifying potassium channel currents [36,37]. NPY receptors can also regulate gene transcription by activating CREB (extracellular signal-regulated kinase or cAMP response element-binding protein) signaling [38,39]. Expression of Y1 and Y2 receptors, the two major receptor subtypes, has been observed in the frontal cortex, lateral septum, NAc, bed nucleus stria terminalis (BNST), PVN, lateral hypothalamus, amygdala, hippocampus, nucleus solitary tract, and area postrema [25,40,41]. Y5 receptor is also widely distributed in the brain of the rat. In some regions such as cerebral cortex, caudate putamen, amygdala and PVN, Y5 receptor colocalizes with Y1 receptor [42,43].

NPY regulates multiple physiological and pathophysiological processes involved in food intake, fear and anxiety, learning and memory, depression, posttraumatic stress, and processing of pain and itch [40,44,45,46,47,48,49,50,51,52]. Moreover, the local effect of NPY on neurons of the brain can support neuronal health and function by stimulating the release of nerve growth factors, reducing neuroinflammation, and inducing autophagy and neurogenesis [53,54,55]. These neuroprotective qualities are considered to arise from the roles of NPY in modulation of neuronal physiology through regulation of calcium homoeostasis, neurotransmitter release, and synaptic excitability [56,57]. Accumulating evidence suggests that NPY may provide neuroprotection in neurodegenerative diseases, such as Alzheimer’s disease, Parkinson’s disease, Machado-Joseph’s disease, and Huntington’s disease [25,32,58,59]. However, NPY may be implicated in the disease pathogenesis of amyotrophic lateral sclerosis [60], and elevated NPY levels in the blood of patients correlated with a shorter disease duration [61].

**Figure 1:**
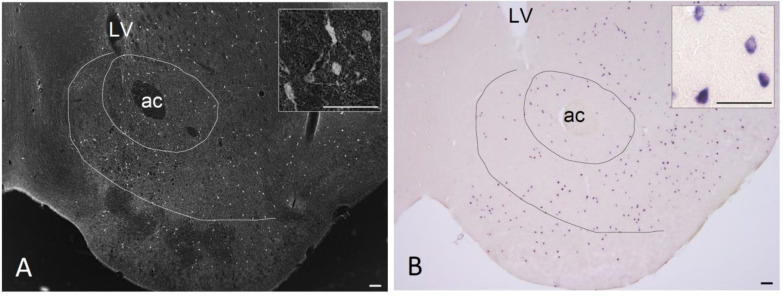
(**A**) Immunohistochemistry of NPY in the NAc of mice. Many medium-sized neurons with fibers are distributed in both the shell and core. Right upper panel shows a region-of-interest at an increased magnification. (**B**) In situ hybridization histochemistry of NPY mRNA by digoxigenin labeling. Dotted lines depict the medial to ventral border of the NAc and boundary between the core and shell. ac, anterior commissure. LV, lateral ventricle. Scale bars: 100 µm in the main figures and 50 µm in right upper panel.

Many reports have demonstrated the orexinergic and anxiolytic properties of NPY with reference to high NPY expression in regions such as the arcuate nucleus, PVN, and amygdala (reviews; [44,45]). In this review, we describe the roles of NPY in the NAc, one of the regions where NPY is expressed most abundantly in the brains of humans and rodents [3,4,5,6,7] (Figure 1). Located in the forebrain, the NAc is the largest component of the ventral striatum (a critical region for reward, motivation, and addiction) and receives mesolimbic dopaminergic innervation.

The NAc can be subdivided at its caudal two-thirds into two different subregions, the shell and the core, each with a different input/output relationship. The shell consists of the peripheral zone of the NAc, whereas the core comprises its central part, surrounding the rostral limb of the anterior commissure [62]. The shell and core can be distinguished by histological and neurochemical profiles, such as cholecystokinin, calbindin, and substance P immunoreactivity [62,63], which are largely paralleled by segregated afferent and efferent connections. Use of anterograde tracers to map efferent projections of the NAc revealed that neurons in the shell preferentially project to the medial ventral pallidum, lateral hypothalamus, ventral tegmental area, substantia nigra pars reticulata, and BNST, whereas neurons in the core primarily project to the dorsal portion of ventral pallidum, medial globus pallidus, and substantia nigra pars compacta [64]. These projection neurons are small to medium in size (medium spiny neurons) [62]. The NAc core and shell receive afferent inputs from various brain regions (more than 50) such as the ventral midbrain, amygdala, hippocampus, and prelimbic and infralimbic cortices [65,66]. In addition, there are direct connections between the core and shell subregions [64,67]. Wide-spread intra-accumbal connections have been observed, including reciprocal projections between specific parts of the shell and core, although fibers originating in the core reach more distant areas of the shell than those projecting from the shell to the core [67].

## 2. Expression of NPY in the NAc

In humans, the NAc is one of the areas of the brain with the highest expression of NPY-like immunoreactivity, in addition to the caudate, putamen, and amygdala [3,8]. In rats, medium-sized NPY immunoreactive neurons with numerous fibers are observed throughout the medial half of the NAc [10]. Intense Y1 receptor-expressing cells in the human NAc have been reported [68]. Ultrastructural analysis revealed NPY immunoreactivity principally in aspiny type neurons that had no detectable spines on their dendrites. NPY-immunoreactive neurons had spindle-shaped cell bodies 15–25 µm in diameter that contained large, multiply indented nuclei and prominent Nissl bodies in rats [69,70]. These presumably intrinsic neurons receive synaptic input from GABAergic terminals [69,70]. NPY-immunoreactive axon terminals contained numerous small clear vesicles and one or more large dense-core vesicles. These terminals target unlabeled somata, as well as labeled and unlabeled distal dendrites. Few synaptic contacts existed between tyrosine hydroxylase (TH)-labeled terminals and NPY-immunoreactive dendrites, although both TH- and NPY-positive terminals converged on the same unlabeled dendrites. Moreover, few axon terminals exhibited both TH immunoreactivity and NPY-immunoreactive dense-core vesicles, suggesting that some dopaminergic afferents from the brain stem coexist with NPY. Notably, these NPY-and-TH-colocalized terminals were found in the NAc, but not the dorsal striatum [69].

Astrocytic processes were observed in close association with NPY-immunoreactive dendrites [69]. Y1 receptors were present in somatodendritic and axonal profiles that contained NPY or apposing spiny neurons. The density of Y1 receptor-immunoreactive dendrites and spines was greater in the motor-associated core than in the shell of the NAc. Moreover, Y1 receptors in the NAc were implicated in both the post- and presynaptic effects of NPY, and affected certain neurons associated with motor control [34]. These ultrastructural observations suggest that aspiny NPY neurons in the NAc receive direct GABAergic inputs, but few dopaminergic inputs, and control projecting neurons through pre- and postsynaptic Y1 receptors.

### Expression of NPY Is Altered by Drug Treatments

The NAc mediates reward-related behavior, and the mesolimbic dopamine system is involved in rewarding effects of addictive drugs [71]. NPY neuron density in the NAc was decreased by unilateral 6-hydroxydopamine (6-OHDA) lesioning of nigral dopaminergic neurons, particularly on the contralateral side. Dopamine depletion induced by exposure to α-methylparatyrosine similarly decreased NPY neuron density in the NAc [72]. The reduction of NPY neurons elicited by 6-OHDA was differentially reversed by apomorphine between the anterior and posterior NAc, suggesting dopamine-dependent and dopamine-independent NPY responses [72]. Repeated administration of psychotomimetic drugs, such as methamphetamine and cocaine, reversibly reduced NPY expression at peptide and mRNA levels in the NAc [73]. This reduction appears to have been mediated through a decrease in NPY biosynthesis in response to changes in mesolimbic dopamine input [74]. NPY immunoreactivity in the NAc was decreased after the administration of noncompetitive NMDA antagonists phencyclidine-HCl and MK801. In addition, the D2/D3 receptor antagonist sulpiride significantly decreased NAc NPY contents [73,75,76]. Two-week administration of neuroleptics such as chlorpromazine, haloperidol, and clozapine decreased NPY contents and mRNA in the NAc, suggesting that their activities are mediated by dopaminergic D1/D2 receptors [76,77,78]. Chronic treatment with anxiolytics, such as diazepam (benzodiazepine family) and buspirone (5-HT1A agonist), decreased NPY levels detected by radioimmunoassay in the NAc [79]. Two-week administration of the 5-HT2A antagonist ketanserine also reduced NPY levels in the NAc [78]. Recently, lesions of the laterodorsal tegmental nucleus, a major external source of cholinergic innervation of the NAc, were shown to decrease the number of NPY-immunoreactive neurons in the NAc [80,81]. Collectively, these studies indicate that dopaminergic, glutamatergic, GABAergic, serotonergic, and cholinergic neuronal inputs regulate NPY expression in the NAc via their respective receptors.

## 3. NPY and Alcohol Intake in the NAc

Previous reports regarding NPY expression in the NAc and the effect of alcohol intake are somewhat confusing because they show both negative and positive associations, probably due to differences in drinking conditions and receptor signaling. Regardless, NPY has a role in alcohol and drug abuse disorders [82,83]. In both NPY-knockout and NPY-overexpressing transgenic mice, NPY expression levels were negatively correlated with innate ethanol preference [84]. This correlation also occurs in the C57BL/6J mouse strain, which has lower basal NPY expression levels in multiple brain regions, including the shell of the NAc, compared to the ethanol non-preferring DBA/2J strain [85,86]. Downstream of cAMP signaling, cAMP-dependent protein kinase A (PKA) and CREB, which regulate NPY gene transcription, are considered to be involved in alcohol drinking behavior [87]. Mice lacking the RIIβ subunit of PKA display increased basal PKA activity [88,89] and increased NPY immunoreactivity in the NAc core [90], which has been implicated in ethanol sensitization [91]. RIIβ-knockout mice also show increased consumption of ethanol and behavioral sensitization to ethanol after repeated injection relative to wild-type mice [92,93]. NPY-knockout mice exhibit this ethanol-induced behavioral sensitization, suggesting that elevated NPY signaling in the NAc core contributes to this sensitization and the Y2 receptor modulates this phenomenon [90]. During chronic ethanol ingestion, rats exhibit an increased number of NPY neurons in the NAc that returns to control levels after withdrawal [94]. Acetaldehyde, the first alcohol metabolite, is involved in the rewarding, addictive, and motivational properties of alcohol intake. Acetaldehyde-intoxication induced by repeated intragastric infusion elicits a reduced number of NPY neurons in the shell of NAc, while NPY neurons increase during the withdrawal phase [95].

In ethanol self-administration model rats, the posterior ventral tegmental area (where dopamine neurons send axons to the NAc) dose-dependently increased ethanol self-administration after the administration of NPY or [Leu31, Pro34]-NPY (NPY Y1 receptor agonist) into the NAc shell, whereas BIBP3226 (selective Y1 receptor antagonist) had the opposite effect [96]. Rats conditioned to self-administer ethanol exhibited a significant increase in NPY-immunoreactive cells and fibers in the NAc shell. These results suggest that NPY plays an important role in modulation of the dopaminergic system in the NAc shell [96]. Crabbe et al. constructed a genetic mouse model of risk for binge-like alcohol drinking (high drinking in the dark, HDID) that was selected for high blood ethanol concentrations [97]. Recently, HDID mice were shown to exhibit the same baseline NPY immunoreactivity as control heterogeneous stock (HS) mice in the NAc, BNST, amygdala, and PVN. However, NPY immunoreactivity was reduced in the NAc (particularly in the shell) of HS mice after ethanol drinking, but not in other brain regions. HDID mice showed a blunted NPY response to alcohol in the NAc compared with HS mice, suggesting a region-specific influence on drinking to intoxication [98]. More recently, Brancato et al. reported that in utero treatment with the cannabinoid Δ9-tetrahydrocannabinol leads to increased alcohol drinking and decreased NPY-positive cells in the limbic regions (including the NAc core and shell) of adolescent offspring. Thus, altered NPY signaling due to prenatal cannabinoid exposure may be involved in the development of alcohol-addictive behaviors [99].

From these reports, NPY expression and signaling in the NAc is involved in regulating alcohol preference and alcohol-induced behavioral responses, including locomotor sensitization, addiction, and withdrawal.

## 4. NPY and Feeding Behavior in the NAc

Eating disorders and substance (e.g., alcohol) abuse coexist at a high rate in humans [100]. Accordingly, there may be similar alterations in endogenous neurochemical systems, such as neurotransmitters and neuropeptides, of the brain [101]. NPY is one of the earliest-recognized and most potent orexigenic neuropeptides acting in the hypothalamus [44]. The NAc is considered to be an important region for appetitive behavior and reinforcement. Injection of antagonists of α-amino-hydroxy-5-methylisoxazole-4-propionic acid (AMPA), kainite, and non-NMDA glutamate receptors in the shell of NAc elicited pronounced food intake [102]. Conversely, injection of AMPA into the shell suppressed deprivation-induced feeding [103]. Administration of the GABA_A_ receptor agonist muscimol and GABA_B_ receptor agonist baclofen into the shell of the NAc induced intense, dose-related feeding without altering water intake [104]. These results indicate that inhibiting local neuronal activity in the shell is involved in stimulating food intake. Injection of muscimol into the NAc shell not only induced a large increase in food intake, but also activated neurons in feeding-related regions such as the lateral hypothalamus, arcuate nucleus, and PVN of satiated rats [105]. This increase in food intake was strongly inhibited by intraventricular injection of specific NPY Y1 and Y5 receptor antagonists [105].

Regarding NPY in the NAc, food deprivation for 48 h and refeeding reportedly induces alterations of NPY concentrations in hypothalamic nuclei, such as the arcuate nucleus, PVN, and lateral hypothalamus (LH), but not in the NAc [106]. Bilateral NPY injection into the perifornical LH of rats, but not injection into the NAc, stimulated ingestion of both preferred sucrose and non-preferred powdered laboratory chow [101].

Using conditioned place preference (CPP) testing, accumbal NPY injection was shown to induce reward-related behavior, but not feeding [101]. These studies suggest that, in contrast to the hypothalamus, NPY in the NAc is not involved in the intake of sucrose or laboratory chow.

**Figure 2:**
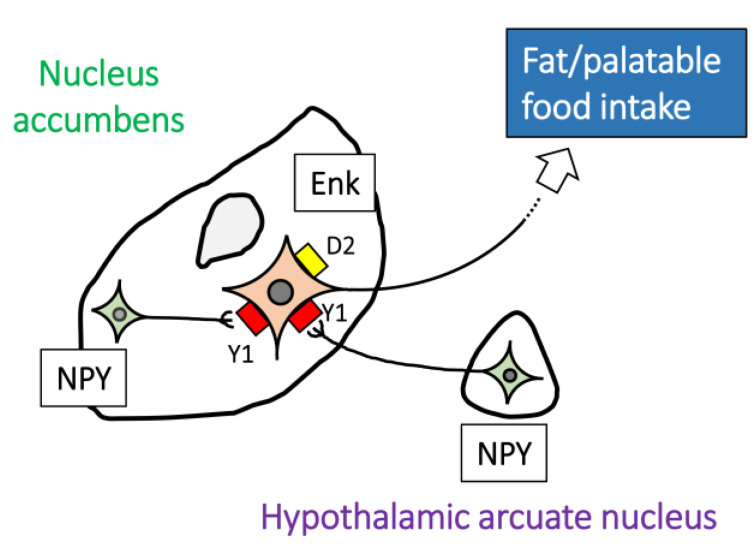
Schematic of NPY-mediated food intake in the NAc. NPY neurons in the NAc or NPY afferents from hypothalamic arcuate nucleus regulate fat/palatable food intake via accumbal enkephalin neurons expressing Y1 and dopamine D2 receptors. Enk, enkephalin neurons; NPY, neuropeptide Y neurons.

Recently, infusion of NPY into the NAc was shown to increase the motivation to respond to a palatable food [107]. Using a free choice of diet, van den Heuvel et al. demonstrated that intra-NAc injection of NPY elicited increased intake of fat, but not sugar or standard chow, which was mediated by the Y1 receptor [108]. This group also showed that intra-NAc injection of NPY reduced neuronal firing, and that NAc-enkephalin neurons expressed Y1 receptor [108]. NPY neurons in the hypothalamic arcuate nucleus project to the NAc and affect hedonic feeding [108,109]. The link between enkephalin neurons, which express D2 receptors, and high-fat food intake has been suggested by a study showing that a diet of highly palatable Ensure (high fat/sugar) affected enkephalin gene expression in the NAc [110]. Furthermore, enkephalin binds to µ-opioid receptors, and intra-NAc administration of the µ-opioid receptor agonist [D-Ala^2^, N-Me-Phe^4^, Gly^5^-ol]-enkephalin produced preferential stimulatory effects on fat ingestion with no effect on carbohydrate ingestion when both high-fat and high-carbohydrate diets were simultaneously present [111]. This selective stimulation of fat intake was suppressed by systemic injection of naltrexone, a general opioid receptor antagonist [111]. Enkephalin in the NAc is expressed in the projecting spiny neurons [112,113]. Taken together, these reports suggest that NPY in the NAc is implicated in intake of fat, but not standard chow, via the Y1 receptor of enkephalin neurons (Figure 2).

## 5. NPY and Reward/Addiction in the NAc

NPY injections in the NAc produce a CPP in rats [114]. These injections may influence dopamine neurotransmission in the NAc because this CPP is blocked by pretreatment of the NAc with the dopamine D1 and D2 receptor antagonist cis-flupenthixol. Application of NPY to the NAc is reportedly involved in rewarding properties mediated by dopamine neurotransmission [114]. Concomitantly, Sørensen et al. reported that NPY infusion into the NAc shell dose-dependently increased extracellular levels of local dopamine, implying accumbal NPY controls reinforcement of addictive drugs [115]. This group also reported that cocaine-induced self-administration, c-Fos expression, and extracellular dopamine in the NAc were attenuated by the Y5 receptor antagonist L-152,804 in Y5-KO mice [116]. These results suggest that the Y5 receptor partly mediates the action of accumbal NPY in dopamine release associated with cocaine-induced behavioral effects. Bilateral intra-NAc shell injection of morphine, NPY, or [Leu^31^,Pro^34^]-NPY (NPY Y1 receptor agonist) increased electrical self-stimulation of the medial forebrain bundle by lever-presses, a measure of reward and reinforcement [117]. In contrast, injection of the selective Y1 receptor antagonist BIBP3226 into the NAc shell had the opposite effect. Furthermore, the reward effect of morphine was significantly potentiated by NPY or [Leu^31^,Pro^34^]-NPY, but antagonized by BIBP3266 [117]. Recently, NPY expression was shown to significantly decrease in the NAc shell immediately after chronic morphine exposure [118]. Subsequently, it rapidly increases and then gradually returns to normal levels. These changes in NPY are thought to be related to morphine-induced CPP and reward memory because injection of NPY into the NAc shell prolonged duration of the extinction period, while blocking of the Y5 receptor in the NAc shell reduced it. Thus, NPY in the NAc may play a role in helping opioid addiction [118].

In a human study, genetically selected subjects at the extremes of high-NPY and low-NPY expression by genotyping had a monetary incentive delay tasks that varied by salience (high versus low) [119]. Functional magnetic resonance imaging revealed that responses of bilateral NAc to high-salience versus low-salience stimuli were greater for low-NPY subjects relative to high-NPY subjects. This finding raises the possibility that individual differences in accumbal NPY expression modulate the risk for addiction and mood disorders [119].

## 6. NPY and Emotional Behavior in the NAc

One of the most studied properties of NPY is its antianxiety action [40,45,120]. However, to date, few studies examining the role of accumbal NPY in anxiety and stress compared these effects to those of other brain regions, such as the amygdala and hypothalamus [40,45,121,122]. NPY mRNA expression in the NAc and hippocampus of Flinder-sensitive line rats, a genetic animal model of depression, was significantly decreased compared to control Flinder-resistant line rats [123]. Frightened rats that were previously subjected to electric foot-shock exhibited increased NPY immunoreactivity in the NAc, amygdala, and hypothalamus with increased numbers of defecation and gastric ulcers [124]. The antianxiety drug diazepam (benzodiazepine receptor stimulant) reversed fear-induced increases in NPY expression in these regions [124]. These results suggest that endogenous NPY in the NAc, as well as other regions, responds to alleviate fear conditioning. Administration of the neuropeptide cholecystokinin (CCK)-4 can induce panicogenic and anxiogenic responses in humans [125]. However, intracerebroventricular (ICV) administration of NPY and Y1 receptor agonists attenuated the anxiety and depression-like effects of CCK-4 ICV administration in mice. NPY-immunoreactive fibers and cell populations were decreased in the NAc shell and ventral part of the lateral division of the BNST [126]. High-fat diet and low-dose streptozotocin induce type 2 diabetes in mice, as well as depression-like behaviors such as increasing immobility time by tail-suspension test [127]. These mice exhibit a significant reduction of NPY immunoreactivity in the NAc, lateral division of BNST, and central nucleus of the amygdala. ICV administration of NPY or the Y1 receptor agonist [Leu^31^,Pro^34^]-NPY and intraperitoneal injection of the antidepressant imipramine decreased immobility time in these mice. However, the opposite effect was observed in response to administration of the Y1 receptor antagonist BIBP32226 ICV [127]. Transgenic mice solely expressing the non-edited 5-HT2C receptor (INI phenotype) exhibited behavioral despair in forced swim testing and significantly decreased 5-HT in the NAc [128]. These mice also exhibited reduced NPY mRNA expression in the NAc, whereas NPY overexpression in the NAc by adeno-associated virus (AAV) injection relieved their depression-like behavior [128]. Together, these results indicate that NPY along with the Y1 receptor in the NAc can elicit an antidepressive effect.

**Figure 3:**
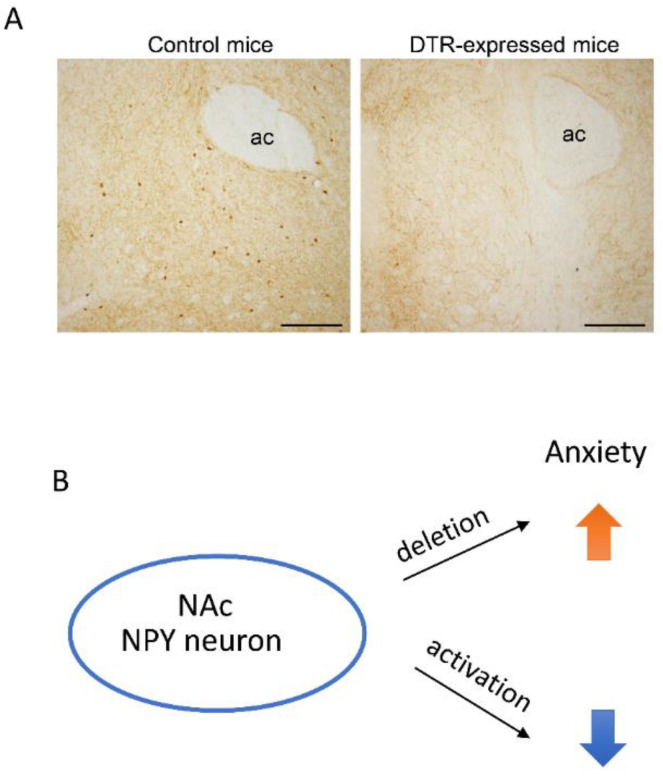
(**A**) NPY immunohistochemistry in the NAc of NPY-Cre mice at 3 weeks after intraperitoneal injection of diphtheria toxin. The right panel shows NPY immunoreactivity of control mCherry-expressing mice. The left panel shows NPY immunoreactivity in DTR-expressing mice. NPY neurons were ablated after diphtheria toxin exposure in NAc-DTR-expressing mice [129]. ac, anterior commissure. Scale bars: 200 µm. (**B**) Schematic showing that when accumbal NPY neurons are deleted, mice exhibit increased anxiety, whereas activation of accumbal NPY neurons caused mice to exhibit anxiolytic behavior [129]. DTR, diphtheria toxin receptor; NAc, nucleus accumbens; NPY, neuropeptide Y.

Recently, we used NPY-Cre mice and AAV injection to specifically ablate or activate NAc NPY neurons to their role in modulating anxiety behavior [129]. Specific deletion of accumbal NPY neurons by Cre-dependent diphtheria toxin receptor expression in the NAc and systemic diphtheria toxin treatment resulted in anxiety behaviors, as indicated by open field and elevated plus maze tests. However, specific activation of accumbal NPY neurons using designer receptors exclusively activated designer drugs (DREADD) technology, producing anxiolytic behaviors in mice according to these tests [129] (Figure 3). These findings suggest that NPY in the NAc is involved in anxiolytic and anti-depressive behavioral effects.

## 7. Other Functions

NPY in the NAc has also been implicated in other functions. Roles of NPY in the modulation of pain processing have been well-studied at the level of spinal cord and dorsal root ganglia [130,131]. Regarding accumbal NPY and pain control, intra-NAc administration of NPY induced dose-dependent increases in hindpaw withdrawal latency to thermal and mechanical stimulation in rats. This anti-nociceptive effect of NPY is blocked by subsequent exposure of the NAc to a Y1 receptor antagonist. This anti-nociceptive effect of accumbal NPY was attenuated by intra-NAc administration of the opioid antagonist naloxone, suggesting that opioid systems are involved in NPY-induced anti-nociception in the NAc [132].

NPY has been implicated in neurodegenerative diseases such as Alzheimer’s disease, Huntington’s disease, and Parkinson’s disease as a biomarker, neuroprotective factor, or potential therapeutic target [25,32,58,133]. Numbers and densities of NPY mRNA-expressing cells in the NAc, caudate nucleus, and putamen were analyzed in healthy individuals and patients with Parkinson’s disease by in situ hybridization histochemistry [134]. In patients with Parkinson’s disease, both the number and silver grain density of NPY mRNA-expressing cells were increased in the NAc and ventral part of the caudate nucleus and putamen. These results suggest that a loss of dopaminergic tone affects NPY neurons of the ventral striatum [134].

Aging also influences NPY expression in the NAc. In aged (24-month-old) rats, numbers of NPY neurons were reduced by 20%, but their size was unaltered. Notably, ICV administration of nerve growth factor to aged rats reversed the age-related decrease in number of NPY neurons [135].

## 8. Conclusions

We have summarized the role of NPY in the NAc by describing its morphology and previously reported features. NPY is expressed mainly in aspiny intrinsic neurons throughout the NAc. Accumbal NPY has been implicated in alcohol intake, drug addiction, food intake, anxiety, and depression, as suggested by studies of alterations in NPY expression by intra-NAc injections of NPY or receptor agonists/antagonists. The control of mesolimbic dopaminergic release elicited by NPY receptors may take part in these functions. NPY in the NAc may exert its physiological and pathophysiological actions partly through neuroendocrine mechanisms and the autonomic nervous system. In the future, the development of drugs capable of modulating NPY expression and NPY receptors in the NAc may contribute to the treatment of alcoholism, drug addiction, eating disorders, anxiety, and depression.
